# Identification of ruptured intracranial aneurysms using the aneurysm-specific prediction score in patients with multiple aneurysms with subarachnoid hemorrhages- a Chinese population based external validation study

**DOI:** 10.1186/s12883-022-02727-w

**Published:** 2022-06-01

**Authors:** Xue-hua Zhang, Xiao-yan Zhao, Lan-lan Liu, Li Wen, Guang-xian Wang

**Affiliations:** 1 Department of Radiology, Banan People’s Hospital, Chongqing Medical University, Chongqing, 400037 China; 2 Department of Radiology, Army Medical University Xinqiao Hospital, Chongqing, 400037 China

**Keywords:** Multiple intracranial aneurysms, Subarachnoid hemorrhage, Risk factors, Computed tomography arteriography, Predictive scoring model

## Abstract

**Background:**

For patients with aneurysmal subarachnoid hemorrhages (SAHs) and multiple intracranial aneurysms (MIAs), a simple and fast imaging method that can identify ruptured intracranial aneurysms (RIAs) may have great clinical value. We sought to use the aneurysm-specific prediction score to identify RIAs in patients with MIAs and evaluate the aneurysm-specific prediction score.

**Methods:**

Between May 2018 and May 2021, 134 patients with 290 MIAs were retrospectively analyzed. All patients had an SAH due to IA rupture. CT angiography (CTA) was used to assess the maximum diameter, shape, and location of IAs to calculate the aneurysm-specific prediction score. Then, the aneurysm-specific prediction score was applied to RIAs in patients with MIAs.

**Results:**

The IAs with the highest aneurysm-specific prediction scores had not ruptured in 17 (12.7%) of the 134 patients with 290 MIAs. The sensitivity, specificity, false omission rate, diagnostic error rate, and diagnostic accuracy of the aneurysm-specific prediction score were higher than those of the maximum diameter, shape, and location of IAs.

**Conclusions:**

The present study suggests that the aneurysm-specific prediction score has high diagnostic accuracy in identifying RIAs in patients with MIAs and SAH, but that it needs further evaluation.

**Supplementary Information:**

The online version contains supplementary material available at 10.1186/s12883-022-02727-w.

## Background

Subarachnoid hemorrhage (SAH) caused by a ruptured intracranial aneurysm (RIA) has high mortality and disability rates [1]. RIAs should be treated as soon as possible to prevent rebleeding, and the choice of treatment method (microsurgical clipping or endovascular coiling) depends on the site of the RIA [2]. Approximately 30% of patients with intracranial aneurysms (IAs) have multiple IAs (MIAs) [3], and approximately one-third of MIAs have uncertain rupture sources [1]. Misdiagnosis of the location of the RIA may lead to postoperative rebleeding and a poorer outcome [4, 5]. Therefore, it is of great clinical value to accurately determine the RIA in MIAs if all IAs cannot be treated at the same time.

The hemorrhage pattern is generally the primary indicator of RIA; however, it is quite difficult to judge RIAs by diffuse or symmetrical bleeding [6]. Although high-resolution contrast-enhanced magnetic resonance vessel wall imaging helps to identify the site of RIA in patients with MIAs, scan time and spontaneous motion are notable limitations [7]. Some scholars used the population, hypertension, age, size, earlier subarachnoid hemorrhage, aneurysm site (PHASES) score and unruptured intracranial aneurysm treatment score (UIATS) to predict RIA [8–11]. However, all these studies showed that the PHASES score and UIATS had a lower ability to identify RIA.

Recently, Hadjiathanasiou et al. [6] developed a novel prediction score, the aneurysm-specific prediction score, for simple and quick identification of RIAs. Encouragingly, the prediction score correctly identified the RIA in all the patients. However, it is not clear whether this score is highly applicable to the Chinese population. After all, in terms of genetics, the Chinese and Caucasian are not identical. Hence, we sought to identify whether the aneurysm-specific prediction score is able to predict RIA in the Chinese population.

## Methods

### Patient population

**Fig. 1:**
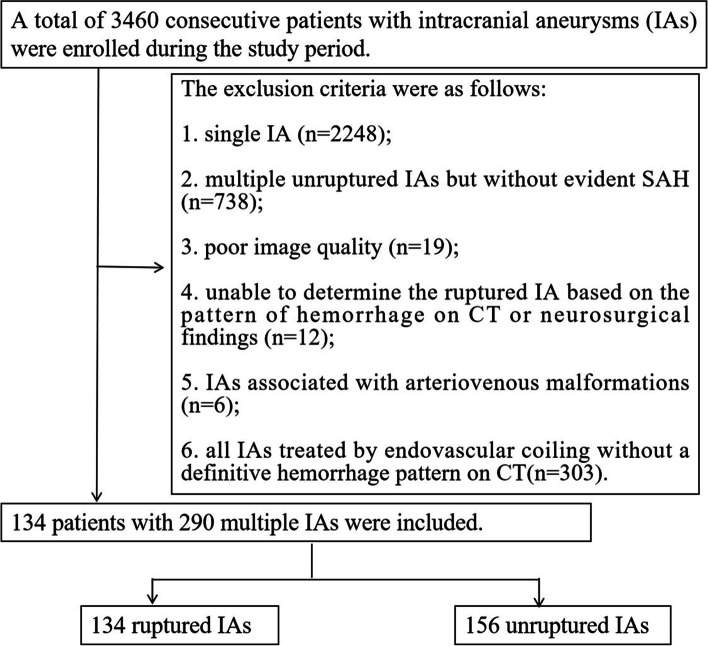
Flow chart of the inclusion process for patients with multiple intracranial aneurysms

This retrospective study was approved by the local ethics committee (Banan People’s hospital, 2,021,015; Xinqiao hospital, 2,016,031), which waived the requirement for informed consent from patients. Between May 2018 and May 2021, at two participating centers, consecutive patients with aneurysmal SAH and more than one IA on CTA were included. SAH was diagnosed by nonenhanced CT or lumbar puncture. RIAs were confirmed in two ways: microsurgical clipping or endovascular coiling. In endovascular coiling, RIAs were identified according to hemorrhage pattern or further CT follow-up. The exclusion criteria were as follows: (1) single IA; (2) multiple unruptured IAs but without evident SAH; (3) poor image quality making it impossible to evaluate the geometric morphology of IAs; (4) inability to determine the RIA based on the pattern of hemorrhage on CT or neurosurgical findings; (5) IAs with vascular malformations; and (6) all IAs were treated by endovascular coiling without a definitive hemorrhage pattern on CT. The patient inclusion flow chart is shown in Fig. 1.

### Imaging protocol and analysis

All patients underwent pretreatment nonenhanced CT and CTA on a 320 multidetector (Toshiba Aquilion One; Toshiba Medical Systems, Tokyo, Japan) or 64 multidetector (GE LightSpeed VCT; GE Healthcare, Milwaukee, Wisconsin, USA) machine. The CTA data were reconstructed with a thickness of 0.5 mm or 0.625 mm and postprocessed to generate three-dimensional volume-rendered images.

All images were analyzed by two experienced radiologists (one with 5 years of experience in neuroradiology and the other with 15 years of experience in vascular imaging), who measured the maximum diameter of IAs and determined their shape and location independently. IA shapes were classified as regular or irregular, with lobular aneurysms or aneurysms with a bleb classified as irregular [6]. IA location is divided into five regions: anterior cerebral artery (AA), including anterior communicating artery (AcomA), internal carotid artery (ICA) excluding posterior communicating artery (PcomA), PcomA, middle cerebral artery (MCA) and posterior circulation (PC) [6]. Maximum diameter was defined as the largest measurement in terms of maximum dome diameter or width [11]. For categorical data, controversial cases were resolved through discussion, and the average values of the continuous data obtained by the two readers were used for analysis.

The maximum diameter, shape and location of IAs were used to calculate the aneurysm-specific prediction score, which is equal to A + B + C: A = 0.0427 × maximum diameter (mm); B = 0 if the IA was located at AcomA and AA, − 0.0104 if located at PcomA, − 0.1831 if located at posterior circulation, − 0.4055 if located at MCA, − 0.5973 if located at ICA; C = 0 if the shape is defined as regular, or 0.5387 if shape is defined as irregular. The aneurysm-specific prediction score was derived from a component-wise gradient boosting algorithm with linear base learners, whose main advantage is the algorithmic procedure of fitting the logistic model (i.e., to estimate its coefficients) [6]. For each patient, the IA with the maximum aneurysm-specific prediction score was predicted as the one that would rupture.

### Statistical analysis

SPSS version 17.0 (SPSS Inc., Chicago, IL, USA) was used for all statistical analyses, and a *P* value less than 0.05 was regarded as statistically significant. The agreement between two observers for the shape and location of the IAs was evaluated by a kappa value. Categorical data and continuous data are expressed as the number of IAs (%) and mean ± standard deviation, respectively. Categorical data were compared by using the chi-squared test, while continuous data were compared using the independent-samples Student’s t test for normally distributed data or the Mann–Whitney U test for nonnormally distributed data. A receiver operating characteristic (ROC) curve was generated to determine the area under the curve.

## Results

One hundred and thirty-four patients with 290 MIAs (one ruptured and the other unruptured) were available for analysis (supplementary file). Among the 33 males and 101 females, the mean ages were 59.5 years for all patients, 54.7 years (range, 41–79 years) for males, and 60.9 years (range, 33–86 years) for females. There were 115 patients with 2 IAs, 16 patients with 3 IAs and 3 patients with 4 IAs.

Interobserver agreement on the CTA categorical factors was good (k = 0.951 for the shape of the IAs, k = 1.000 for the location of IAs). Table 1 summarizes the morphological characteristics of the IAs. The mean maximum diameter was 6.34 ± 3.07 mm (range, 1.8–20.7 mm). The mean aneurysm-specific prediction score was 0.28131 ± 0.48 (range, -0.49909–1.24993). The maximum diameter, irregular shape, location in the PcomA and ICA, and aneurysm-specific prediction score were significantly different between the ruptured and unruptured groups.

**Table 1:** Morphological characteristics of the aneurysms

Morphological characteristics	Unruptured group (*n* = 156)	Ruptured group (*n* = 134)	*P*
Maximum diameter (mm)	5.02 ± 2.44	7.88 ± 3.02	< 0.001
Shape			< 0.001
Irregular	36 (23.1%)	95 (70.9%)	
Regular	120 (76.9%)	39 (29.1%)	
Location			
PC	10 (6.4%)	5 (3.7%)	0.427
AcomA + AA	16 (10.3%)	24 (17.9%)	0.063
PcomA	38 (24.4%)	63 (47.1%)	< 0.001
MCA	52 (33.3%)	33 (24.6%)	0.121
ICA	40 (25.6%)	9 (6.7%)	< 0.001
Aneurysm-specific prediction score	0.03631 ± 0.40	0.56653 ± 0.40	< 0.001

*PC* Posterior circulation, *AcomA* Anterior communicating artery, *AA* Anterior cerebral artery, *ICA* Internal carotid artery, *MCA* Middle cerebral artery, *PcomA* Posterior communicating artery

**Fig. 2:**
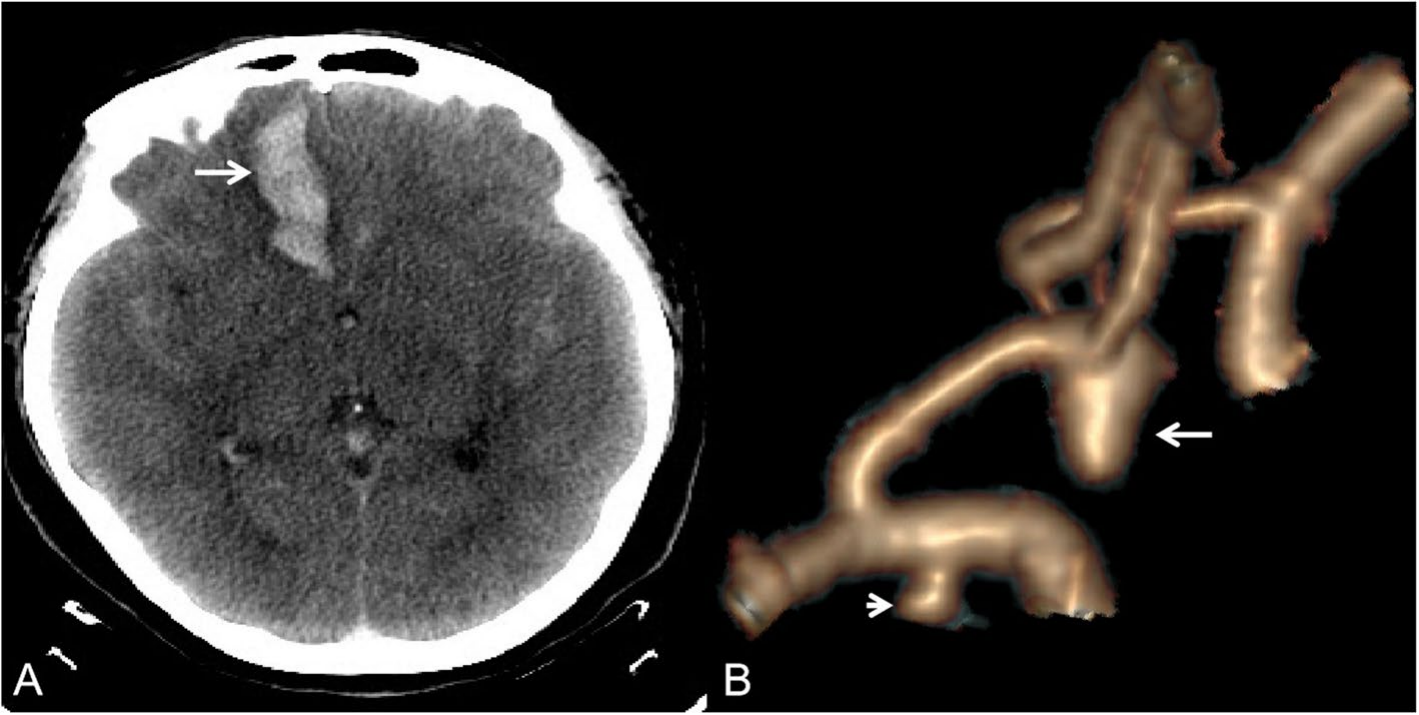
A 52-year-old female presented with severe headache. CT scan showed subarachnoid hemorrhage with a focal hematoma (arrow). Computed tomography angiography showed a ruptured anterior communicating artery aneurysm (large arrow, aneurysm-specific prediction score = 0.90592) and a unruptured internal carotid artery aneurysm (small arrow, aneurysm-specific prediction score = -0.42223)

**Fig. 3:**
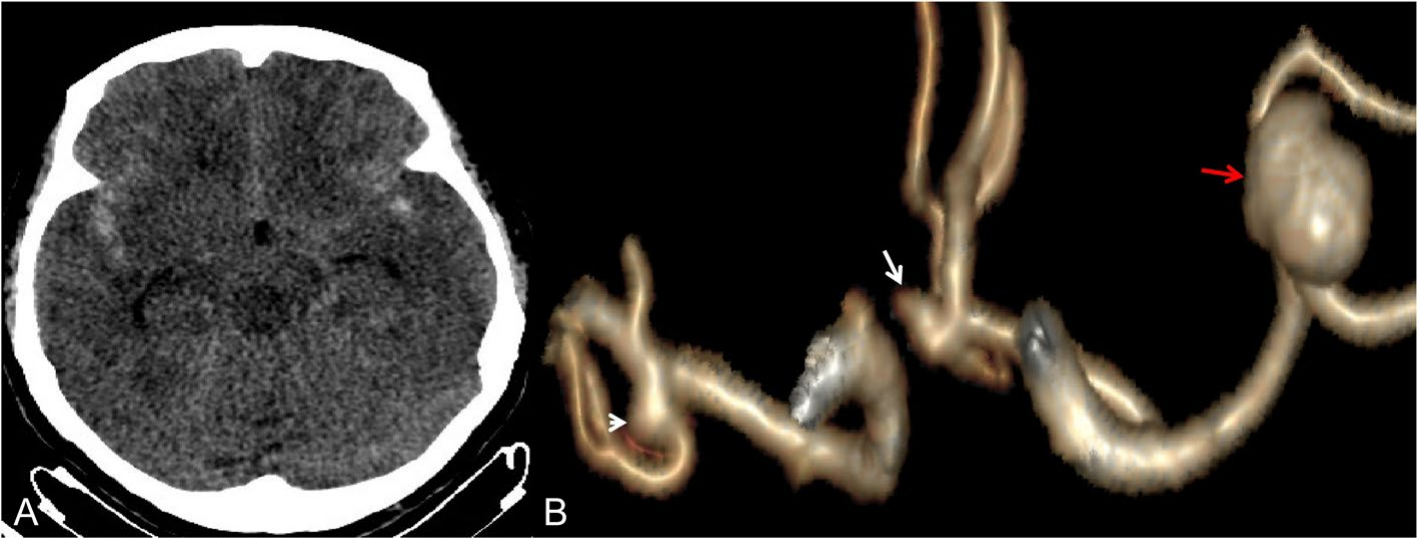
A 62-year-old female presented with symmetrical subarachnoid hemorrhage. Computed tomography angiography showed three IAs located at the left middle cerebral artery (red arrow, ruptured, aneurysm-specific prediction score = 0.59009), right middle cerebral artery (small arrow, unruptured, aneurysm-specific prediction score = -0.22189), and anterior communicating artery (lager arrow, unruptured, aneurysm-specific prediction score = 0.73085)

The diagnostic accuracy of the morphological characteristics of the IAs and the aneurysm-specific prediction score are listed in Table 2. When using maximum diameter alone, the sensitivity, specificity, false omission rate, diagnostic error rate, and diagnostic accuracy were 81.3%, 83.9%, 18.7%, 16.0% and 82.8%, respectively. When using irregular shape alone, the sensitivity, specificity, false omission rate, diagnostic error rate, and diagnostic accuracy were 29.1%, 23.0%, 70.8%, 76.9% and 25.7%, respectively. When using IA location alone, the overall diagnostic accuracy was 43.1–62.4%. When using the aneurysm-specific prediction score, the RIAs were misdiagnosed in 17 patients with 38 MIAs (Table 3). Six RIAs had a large maximum diameter, but due to the location and shape of IAs, the aneurysm-specific prediction score was reduced (Figs. 2 and 3). The sensitivity, specificity, false omission rate, diagnostic error rate, and diagnostic accuracy of the aneurysm-specific prediction score were 87.3%, 89.1%, 12.7%, 10.9%, and 88.3%, respectively.

**Table 2:** Diagnostic accuracy of the morphological characteristics of the IAs and the aneurysm-specific prediction score

Morphological characteristics	Results		Total	SEN%	SPE%	β%	α%	DA%
	UIA	RIA						
Maximum diameter (mm)								
Yes	25	109	134	81.3	83.9	18.7	16.1	82.8
No	131	25	156					
Shape								
Irregular	36	95	131	70.9	76.9	29.1	23.1	74.1
Regular	120	39	159					
Location								
PC	10	5	15	3.7	93.6	96.3	6.4	51.4
AcomA + AA	16	24	40	17.9	89.7	82.1	10.3	56.6
PcomA	38	63	101	47.0	75.6	53.0	24.4	62.4
MCA	52	33	85	24.6	66.7	75.4	33.3	47.2
ICA	40	9	49	6.7	74.4	93.3	25.6	43.1
Aneurysm-specific prediction score								
Largest	17	117	134	87.3	89.1	12.7	10.9	88.3
Nonlargest	139	17	156					
Total	156	134	290					

*IA* Intracranial aneurysm, *SEN* Sensitivity, *SPE* Specificity, *β* False omission rate, *α* Diagnostic error rate, *Da* Diagnostic accuracy, *UIA* Unruptured intracranial aneurysm, *RIA* Ruptured intracranial aneurysm, *PC* Posterior circulation, *AcomA* Anterior communicating artery, *AA* Anterior cerebral artery, *ICA* Internal carotid artery, *MCA* Middle cerebral artery, *PcomA* Posterior communicating artery

**Table 3:** The RIAs that were misdiagnosed in 17 patients with 38 MIAs

Patients	Size (mm)	Location	Shape	Aneurysm-specific prediction score	Ruptured
1	7.9	PcomA	Irregular	0.86563	No
	7.6	PcomA	Irregular	0.85282	Yes
2	5.3	PcomA	Regular	0.21591	No
	3.7	PcomA	Regular	0.14759	Yes
3	4.5	AcomA	Irregular	0.73085	No
	4.3	MCA	Regular	-0.22189	No
	10.7	MCA	Irregular	0.59009	Yes
4	2.4	PcomA	Irregular	0.63078	No
	4.8	PcomA	Regular	0.19456	Yes
5	4.7	PcomA	Irregular	0.72899	No
	4.0	PcomA	Regular	0.1604	Yes
6	6.9	MCA	Irregular	0.42783	No
	4.1	MCA	Regular	-0.23043	Yes
7	7.5	AA	Regular	0.32025	No
	6.1	AcomA	Regular	0.26047	Yes
8	6.1	MCA	Irregular	0.39367	No
	8.4	ICA	Regular	-0.23862	Yes
9	7.3	MCA	Irregular	0.44491	No
	3.7	PcomA	Regular	0.14759	Yes
10	4.3	PcomA	Regular	0.17321	No
	5.3	ICA	Regular	-0.37099	Yes
11	7.4	AcomA	Irregular	0.85468	No
	14	ICA	Irregular	0.5392	Yes
12	5.1	AcomA	Regular	0.21777	No
	3.6	MCA	Regular	-0.25178	Yes
13	5.4	PcomA	Regular	0.22018	No
	11.6	MCA	Regular	0.08982	Yes
14	3.3	ICA	Regular	-0.45639	No
	3	ICA	Regular	-0.4692	Yes
15	6.8	MCA	Irregular	0.42356	No
	5.4	PcomA	Irregular	0.75888	No
	6.7	PcomA	Regular	0.27569	Yes
16	7	PcomA	Irregular	0.8272	No
	5.7	PcomA	Irregular	0.77169	Yes
17	2.8	MCA	Regular	-0.28594	No
	7.1	PcomA	Irregular	0.83147	No
	3	ICA	Regular	-0.4692	No
	6.6	PcomA	Irregular	0.81012	Yes

*RIAs* Ruptured intracranial aneurysms, *MIA* Multiple intracranial aneurysms, *AA* Anterior cerebral artery, *AcomA* Anterior communicating artery, *ICA* Internal carotid artery, *PcomA* Posterior communicating artery, *MCA* Middle cerebral artery

**Fig. 4:**
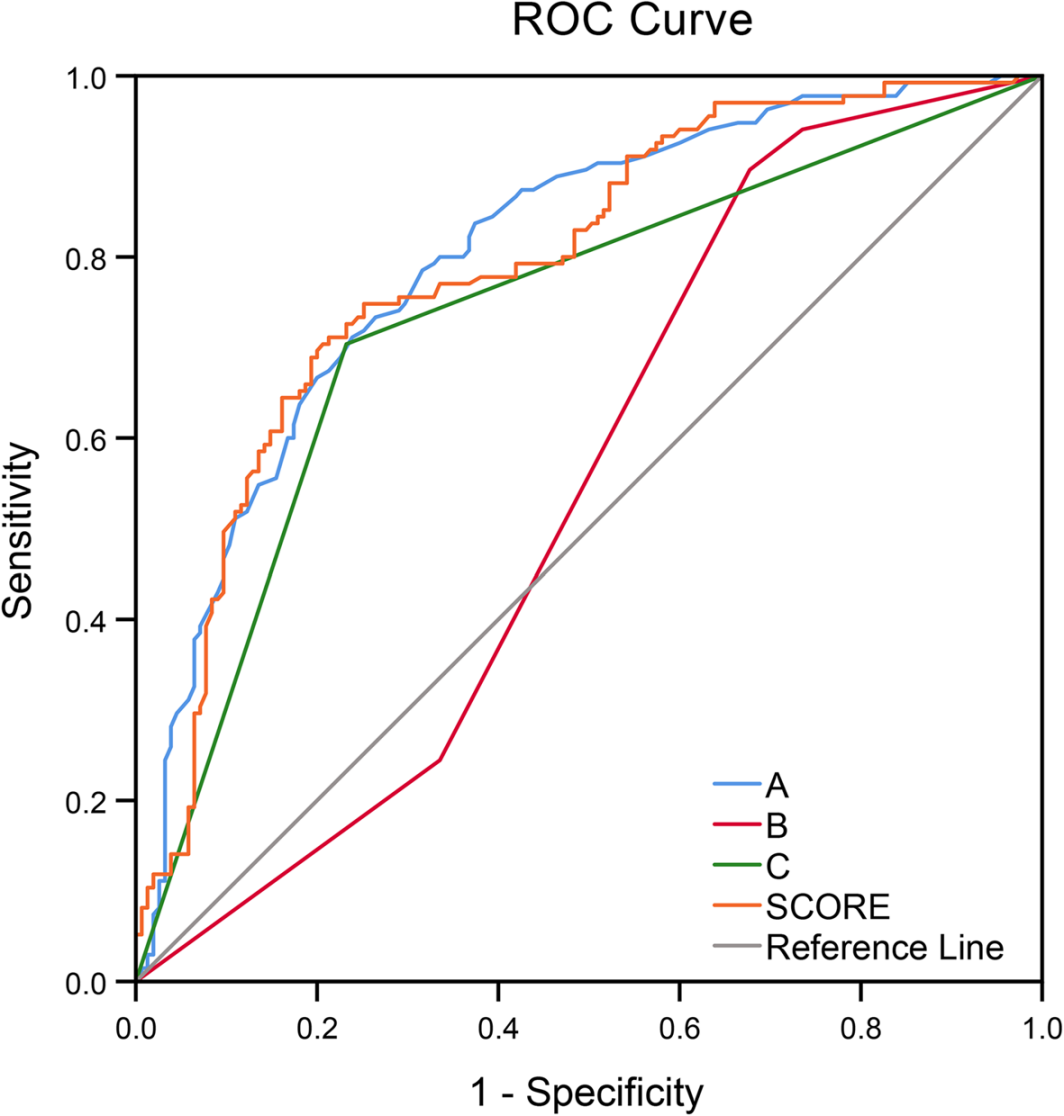
Area under the receiver operating characteristic curve values for **A** (size, 0.798; 95% confidence interval, 0747–0.849), **B** (location, 0.536; 95% confidence interval, 0.468–0.603), **C** (shape, 0.736; 95% confidence interval, 0.677–0.795) and aneurysm-specific prediction score (0.781; 95% confidence interval, 0.724–0.834)

The ROC analysis was performed for continuous data. The areas under the curve for maximum diameter, location, shape and the aneurysm-specific prediction score were 0.798, 0.536, 0.736 and 0.781, respectively (Fig. 4 and Table 4).

**Table 4:** Area under the curve analysis for A, B, C and the aneurysm-specific prediction score

Characteristic	Area	*P*	95% confidence interval
A	0.798	< 0.001	0.747–0.849
B	0.536	0.297	0.468–0.603
C	0.736	< 0.001	0.677–0.795
Aneurysm-specific prediction score	0.781	< 0.001	0.728–0.834

A, size = 0.0427 × maximum diameter of aneurysm (mm); B, location = 0, − 0.0104, − 0.1831, − 0.4055, or − 0.5973; C, shape = 0 or 1

## Discussion

The aneurysm-specific prediction score was established according to IA size, location and shape and was developed to identify RIAs in SAH patients harboring MIAs [6]. In this study, we applied the aneurysm-specific prediction score in 134 SAH patients with MIAs and found that the sensitivity, specificity, false omission rate, diagnostic error rate, and diagnostic accuracy were 87.3%, 89.1%, 12.7%, 10.9%, and 88.3%, respectively.

Traditionally, size has been considered an important factor in IA rupture, and a large IA is considered more prone to rupture than a small IA. Some studies have reported that size is a significant predictive factor for IA rupture [12, 13]. Although Björkman et al. [14] indicated that IA size was associated with IA rupture, the RIA was not of the largest size in 13% of their study cohort, and they found that irregular shape may identify the RIA better than size in patients presenting with SAH and MIAs. In addition, Backes et al. [2] reported that RIA was not the largest IA in 29% of patients with MIAs. In this study, 18.7% (25/134) of the patients had an unruptured IA with the largest diameter, and 15 of them did not have the largest aneurysm-specific prediction score.

Irregular shape was thought to be associated with IA rupture [12, 13], possibly because the irregular shape increases the local hemodynamic stress [15]. Backes et al. [2] reported that irregular shape is associated with IA rupture independent of IA size and location and independent of patient characteristics. Björkman et al. [14] showed that shape and size had the best diagnostic value for identifying RIAs in patients presenting with SAH and MIAs, but shape may be better than size. However, Orning et al. [4] reported that it is unreliable to use morphological features of IA in determining rupture sites in nondefinitive SAH patterns. Another study also showed that morphological and hemodynamic parameters seem to have no or only low effect on the prediction of RIA in patients with MIAs [16]. The present results showed that 39 (29.1%) RIAs had regular shapes, and 36 (23.1%) unruptured IAs had irregular shapes.

IAs located in the AcomA, PcomA, or PC are considered to have a high risk of rupture [17–19]. The American Heart Association/American Stroke Association indicated that the treatment decision regarding UIAs is based mainly on the size and location [20]. In this study, PcomA, AcomA and MCA were the most common sites in aneurysmal SAH patients. These results are consistent with previous study [21]. Although IAs located in the PcomA ruptured more often than IAs in other locations, the diagnostic accuracy was only 62.4%.

The aim in developing the aneurysm-specific prediction scoring system was to identify RIAs in SAH patients with MIAs, and the prediction score had high accuracy in a small prospective sample [6]. In this study, the aneurysm-specific prediction score had high sensitivity and specificity, but 17 UIAs were misdiagnosed as RIAs. On the other hand, the area under the curve of the aneurysm-specific prediction score was lower than that of maximum diameter, indicating that the performance of the aneurysm-specific prediction score was not satisfactory. One of the reasons is that IA size and shape may change after rupture. Another reason is the inherent flaws of the aneurysm-specific prediction scoring system: sometimes the location and shape of IAs may lead to a decrease in the aneurysm-specific prediction score. The coefficients need to be optimized to further improve the rate of recognition of RIAs. In addition, morphological characteristics such as location of bifurcation, small-diameter of the parent artery, and location of the AcomA with A1 dominance are risk factors for IA rupture [22, 23]. Some studies reported that an aspect ratio ≥ 1.3 or the size ratio were the best factor for identifying RIAs [2, 24]. Finally, different populations may lead to different results. It is well known that Japanese and Finnish patients have a higher risk of IA rupture than those from other geographic regions [25]. While, a nationwide epidemiological in China showed that among the patients with aneurysmal SAH, only 15.4% had MIAs [21], which less than Caucasian and Japanese population [17, 26].

### Limitations

The present study had a limitations. First, the shape or size of the RIAs might have changed due to the rupture, and the results may be biased. Second, this study considered only MIAs with SAH, and the results may not be applicable to patients with a single IA or unruptured MIAs. Third, as we used CTA data in this study, conus arteriosus could have been misdiagnosed as an IA, causing a patient with a single real IA to be identified as one with “MIAs”, although this situation is unlikely. Fourth, the sample size is relatively small in this study, half of the size of the originally published cohort by Hadjiathanasiou et al. [6]. Last, this study only validated the accuracy of the aneurysm-specific prediction scoring system and did not compare it with other scoring systems. A multicenter prospective study with a large sample size is needed in the future.

## Conclusions

We applied the aneurysm-specific prediction score to Chinese patients with MIAs and SAH to identify RIAs and found that the scoring system had high diagnostic accuracy but was not perfect. Larger cohorts for prospective evaluation are warranted in the future.

## Supplementary Information

**Additional file 1.**

## Data Availability

All data generated or analysed during this study are included in this published article and its supplementary information files.
